# Temperature and Aging Affect Glyphosate Toxicity and Fatty Acid Composition in *Allonychiurus kimi* (Lee) (Collembola)

**DOI:** 10.3390/toxics9060126

**Published:** 2021-05-31

**Authors:** June Wee, Yun-Sik Lee, Yongeun Kim, Jino Son, Kijong Cho

**Affiliations:** 1 Department of Environmental Science and Ecological Engineering, Korea University, Seoul 02841, Korea; dnlwns@korea.ac.kr; 2 O-Jeong Eco-Resilience Institute, Korea University, Seoul 02841, Korea; ssamppong@korea.ac.kr (Y.-S.L.); kyezzz@korea.ac.kr (Y.K.); 3 Biological and Genetics Resources Assessment Division, National Institute of Biological Resources, Incheon 22689, Korea; jinoson@gmail.com

**Keywords:** herbicide, degradation, microorganism, risk assessment, arachidonic acid

## Abstract

Glyphosate is the most used herbicide worldwide, but enormous use of glyphosate has raised concerned about its environmental loadings. Although glyphosate is considered non-toxic, toxicity data for soil non-target organisms according to temperature and aging are scarce. This study examined the toxicity of glyphosate with the temperature (20 °C and 25 °C) and aging times (0 day and 7 days) in soil using a collembolan species, *Allonychiurus kimi* (Lee). The degradation of glyphosate was investigated. Fatty acid composition of *A. kimi* was also investigated. The half-life of glyphosate was 2.38 days at 20 °C and 1.69 days at 25 °C. At 20 °C with 0 day of aging, the EC_50_ was estimated to be 93.5 mg kg^−1^. However, as the temperature and aging time increased, the glyphosate degradation increased, so no significant toxicity was observed on juvenile production. The proportions of the arachidonic acid and stearic acid decreased and increased with the glyphosate treatment, respectively, even at 37.1 mg kg^−^^1^, at which no significant effects on juvenile production were observed. Our results showed that the changes in the glyphosate toxicity with temperature and aging time were mostly dependent on the soil residual concentration. Furthermore, the changes in the fatty acid compositions suggest that glyphosate could have a chronic effect on soil organisms.

## 1. Introduction

Glyphosate-based herbicides are systemic herbicides widely used worldwide, and are known to be environmentally benign [1]. This is because glyphosate is known not only to be safe for non-target species [2], but also to be rapidly degraded by microorganisms under environmental conditions [3]. Along with these properties, the introduction of transgenic glyphosate-resistant crops (GRCs) has made glyphosate the most popular and effective herbicide [1]. However, the rapid increase in the use of glyphosate has raised concerns about its environmental loadings [4]. In particular, glyphosate soil residues have been a major concern, because a significant amount of glyphosate can reach the soil, despite its foliar application [5]. Silva et al. (2018) [6] reported that glyphosate was found at concentrations of up to 2 mg kg^−1^ in 45% of topsoil in Europe, even where GRCs are not cultivated. Furthermore, in Argentina, where GRCs have been intensively cultivated, glyphosate was found in all of the investigated farmland soils, with a peak concentration of 8.11 mg kg^−1^ [7]. Because of the increase in glyphosate-resistant weed species [8], the glyphosate application amount and frequency are subsequently increasing [4], suggesting strongly that the actual glyphosate concentrations in soil could be much higher than those measured or estimated in previous studies.

Glyphosate in soil might have a significant effect on soil organisms that are constantly in contact with soil particles. To date, most studies on the impact of glyphosate on the soil ecosystem have focused on soil microorganisms [9,10] because of its unique mode of action via biochemical pathways that only exist in some microorganisms and green plants that utilize the shikimate pathway containing the 5-enolpyruvyl-3-shikimate phosphate synthase (EPSPS) enzyme, which is the target of glyphosate [1]. However, recent studies have reported that soil invertebrates that do not have the EPSPS pathway can also be affected by glyphosate [11,12,13]. Santos et al. (2010) [12] reported that the reproduction of *Folsomia candida* (Collembola) is negatively affected by glyphosate exposure. At the same time, many studies have reported conflicting results, showing no negative effects on the survival rate and reproduction of soil animals [2,14]. Still, there is no general agreement on the toxicity of glyphosate to soil animals, and further studies are needed to clarify this.

The glyphosate toxicity to soil animals can change depending on the environmental factors and exposure duration in the soil. Pesticides entering the soil can be degraded, adsorbed to soil particles, or transported to other media depending on their physicochemical properties [15]. During these processes, the availability of certain chemicals decreases over time, which is referred to as aging [16]. The aging time is a critical determinant of toxicity, because it is directly related to the actual concentration at which soil organisms are exposed. In the case of glyphosate, which easily reacts with soil particles and rapidly degrades, the effect of aging time on toxicity can be profound even within a short duration. The aging effects of pesticides on organisms can vary depending on environmental factors, such as moisture, soil properties, and temperature [16]. Temperature is a key factor in determining not only the fate of pesticides in the environment, but also the response of organisms exposed to pesticides [17]. Because temperature is closely related to the reaction rate of a chemical, its change can modify the fate of pesticides, such as their degradation and adsorption. For example, many studies have reported that the increase in temperature accelerates the degradation of pesticides owing to the enhancement of microbial activity [17,18]. In addition, depending on the exposure temperature, the responses of organisms to pesticides, such as their feeding activity, detoxification, and metabolic rate, can change [19]. Therefore, understanding the effects of aging on the toxicity of glyphosate in relation to temperature is important for assessing its impact on soil animals. Many studies have focused on the effects of aging and temperature on the fate of glyphosate in soil [20,21], but not so much on the effects of these two factors on toxicity.

Among the soil organisms, collembolans have been used as an international standard species to assess soil toxicity [22], because they play a key role in the functioning of the soil environment and are sensitive to various soil contaminants [13,23,24]. The standard toxicity procedure using collembolan species investigates adult mortality and juvenile production as endpoints to assess the impact of contaminants on the soil ecosystem. Although these two endpoints provide valuable information for evaluating toxicity in the soil, the results of bioassays performed under standardized experimental conditions are often difficult to apply to field toxicity assessments, because they do not include variations in environmental factors such as temperature, precipitation, and soil texture occurring in the field. In addition, the results are difficult to apply if the response of the organism is observed only at the sub-individual level. Biomarker-based approaches have been extensively applied to assess these sub-individual effects [25,26]. Enzyme activity [27], protein composition [28], and gene expression [29] have been mainly used as toxicity biomarkers for soil animals. In addition to these biomarkers, fatty acids are also considered potentially important biomarkers, because they are essential components of organisms and play a critical role in energy storage, cell structure, and regulatory physiology [30]. Despite these important properties, few studies have investigated the effects of changes in the fatty acid composition of collembolan species after exposure to toxic chemicals.

In this study, *Allonychiurus kimi* (Lee, 1973) (Collembola: Onychiuridae), which is listed as an alternative test species for *F. candida* [22], was used as a model species. *A. kimi* has been used to evaluate the toxicity of various chemicals, including heavy metals and organic contaminants [23,31]. The effects of temperature on the biology of *A. kimi* have also been thoroughly investigated [32]. This species is also known to be suitable for evaluating soil contaminants using biomarkers [33].

The main objective of this study was to investigate the changes in the toxicity of commercially formulated glyphosate (Geunsami^®^) to *A. kimi* at two temperatures (20 °C and 25 °C) and two aging times (0 day and 7 days). We hypothesized that as the temperature and aging time increase, accelerated degradation of glyphosate would reduce the toxicity to *A. kimi*. To test this hypothesis, the degradation kinetics of glyphosate were investigated with respect to temperature. In addition, the total cellular fatty acid composition of *A. kimi* adults exposed to glyphosate was investigated according to the temperature to determine the toxicity effects at the biochemical level.

## 2. Materials and Methods

### 2.1. Test Animals

The test collembolan species, *A. kimi* (formerly known as *Paronychiurus kimi*), was collected from a paddy field in Korea in 1996 [23]. The *A. kimi* population was cultured in a plastic petri dish (9.0 cm in diameter and 1.5 cm in height) filled with approximately 0.5 cm depth of moist substrate comprised of plaster of Paris, activated charcoal, and distilled water at a ratio of 4:1:4 by volume in a dark growth chamber at 20 ± 1 °C. Brewer’s yeast (Sigma-Aldrich, St. Louis, MO, USA) was provided weekly as food. To obtain age-synchronized collembolans, the eggs laid by hundreds of adults were transferred to a fresh moist substrate using a fine-haired brush. After the eggs hatched, the juveniles were reared under the same conditions and used for all subsequent experiments.

### 2.2. Chemical and Toxicity Test

A commercial formulation of glyphosate (Geunsami^®^) was obtained from Farm Hannong Ltd. (Seoul, Korea) and used throughout the experiments. The formulation contained glyphosate as an active ingredient (41%, *w*/*w*) in the form of isopropylamine salt.

OECD artificial soil was used as the test substrate [22]. The test substrate was prepared by mixing 75% sand, 20% kaolin clay, and 5% sphagnum peat (<2 mm) based on dry weight. Calcium carbonate (Sigma-Aldrich, St. Louis, MO, USA) was added to adjust the pH to 6.0 ± 0.5.

Toxicity tests were conducted according to OECD guideline 232 [22]. The effects of glyphosate on the adult survival and juvenile production of *A. kimi* at 20 °C and 25 °C and aging times of 0 day and 7 days were investigated. The test concentrations were 0.0, 0.7, 3.7, 37.1, 74.1, and 370.5 mg kg^−1^ soil dry weight. The stock solution was prepared by dissolving the formulation in deionized water. Diluted stock solutions adjusted to the test concentrations were mixed thoroughly with the soil to obtain the required moisture content of 50% water holding capacity. The spiked soil (30 g) was placed in a polystyrene vessel (55 mm in diameter and 60 mm in height). A total of 120 test vessels (6 concentrations × 5 replicates × 2 temperatures × 2 aging times) were prepared at once, and 60 test vessels were separately stored in a dark growth chamber at 20 °C and 25 °C, respectively. After 0 day and 7 days of aging at each temperature, 30 test vessels (5 vessels per concentration) were randomly selected and used for the toxicity test.

At the beginning of each test, 10 *A. kimi* adults (42–46 day old) were introduced into each test vessel. The test vessels were kept under the condition that the aging proceeded at each temperature. The test vessels were aerated and weighed weekly to replenish the moisture loss by the addition of deionized water, if needed. Brewer’s yeast was provided on the soil surface as food at the beginning of the test and biweekly. After 28 d, the surviving adults and juveniles produced in each vessel were counted by flotation with water and transferred to a petri dish filled with a moist substrate.

### 2.3. Fatty Acid Analysis

The effects of glyphosate treatment on the fatty acid composition of adults were determined at 20 °C and 25 °C. The test concentrations were 0.0 (control), 37.1, and 370.5 mg kg^−1^. Because no significant effect on adult mortality was observed at any of the glyphosate concentrations, the tested concentrations were selected based on the lowest and highest concentrations at which a decrease in juvenile production was observed.

After 28 day of exposure to 37.1 mg kg^−1^ and 370.5 mg kg^−1^ of glyphosate at 20 °C and 25 °C, the adults that survived the glyphosate treatments were transferred to and kept in the plastic petri dish for 1 d without feeding to excrete their gut contents [34]. The adults exposed to the same concentration were pooled and transferred to a 2 mL microtube and then stored at −80 °C until fatty acid analysis.

The total cellular fatty acids in *A. kimi* adults were analyzed as described by Haubert et al. (2004) [35]. Briefly, 20 adults exposed to the same concentration were homogenized in 7.5 mL of a mixture of chloroform, methanol, and 0.05 M of phosphate buffer at a pH of 7.4 (1:2:0.8, *v*/*v*/*v*). After overnight shaking, 0.8 mL of distilled water and 0.8 mL of chloroform were added. The mixture was centrifuged at 1500 rpm for 5 min and then allowed to stand to separate into three layers of methanol, phosphate buffer, and chloroform. The chloroform layer was then transferred to a new tube. The lipids in the chloroform layer were saponified and methylated using the Sherlock Microbial Identification System (MIDI Inc., Newark, DE, USA) to produce fatty acid methyl esters (FAMEs). Finally, the FAMEs were analyzed using the Sherlock Microbial Identification System consisting of an Agilent 7890A series gas chromatograph equipped with a flame ionization detector. This procedure was repeated three times. In addition, the total cellular lipids (TCLs) in *A. kimi* adults were estimated using an external standard curve of palmitic acid (16:0).

### 2.4. Glyphosate Degradation in Soil

To investigate the temperature-dependent degradation kinetics of glyphosate, 30 g of soil spiked with 370.5 mg kg^−1^ of glyphosate were prepared and stored at 20 °C and 25 °C. The glyphosate concentrations in 5 g of soil were measured at 0, 2, 4, 7, and 14 days after exposure with three replicates each. The control group was treated with distilled water only. In addition, a series of diluted glyphosate solutions (0–20 mg L^−1^) was prepared by dissolving a standard glyphosate solution (1000 μg mL^−1^; Sigma-Aldrich, St. Louis, MO, USA) in 20 mL of 2 M ammonium hydroxide and used to obtain a standard curve.

The glyphosate concentration in the soil samples was determined following the method proposed by Çetin et al. (2017) [36] and Jan et al. (2009) [37] with minor modifications. Briefly, 5 g of soil sample (wet weight) was extracted with 40 mL of 2 M ammonium hydroxide for 24 h. The extracts were centrifuged at 3500 rpm for 3 min to obtain the supernatant. The filtered supernatant (20 mL) was transferred to 50 mL conical tubes. Cu (II) solution (0.1 mL) and ammonia (1 mL) were added to the tubes, followed by gentle shaking. Dispersive liquid (2.5 mL), which consisted of 79.6% acetonitrile, 20.0% dichloromethane, and 0.4% carbon disulfide, was added instantly. After centrifuging for 3 min at 3500 rpm, the dispersed fine droplets of dichloromethane at the bottom of the tube were transferred to a spectrophotometer glass cuvette (Ultrospec 3000 Pro, Pharmacia Biotech, Cambridge, UK). The absorbance of the complex was measured at 435 nm.

A linear relationship between the absorbance and concentration of glyphosate in standard solutions was obtained (*r*^2^ = 0.92). No glyphosate was detected in the control soil, and the recovery rate of glyphosate was 79.59 ± 12.75% in the glyphosate-treated soil. The limit of quantification of glyphosate was 2.08 mg kg^−1^.

### 2.5. Data Analysis

The degradation of glyphosate over time was fitted to a single first-order (SFO) kinetic model [38], as follows:

(1) $$C_{t}=C_{0}e^{-kt},$$

where *C_t_* is the concentration of glyphosate at time *t*, *C*_0_ is the initial concentration of glyphosate (*t* = 0), and *k* is the degradation rate constant. The degradation half-life time (DT_50_), the time required for a 90% decline in concentration (DT_90_), and *k* were estimated by fitting Equation (1) into a non-linear regression model.

To evaluate the effects of the temperature and aging time on the toxicity, an effective concentration of 50% (EC_50_) on juvenile production and the corresponding 95% confidence intervals were estimated by fitting the data to a logistic model [39]. The no observed effect concentration (NOEC) and lowest observed effect concentration (LOEC) on juvenile production were determined by applying one-way analysis of variance (ANOVA), followed by Dunnett’s post hoc test (*p* < 0.05).

Prior to statistical analysis of the fatty acid profile, the data were arcsine-root transformed to normalize the distribution. The unsaturation index (UI; sum of the percentage of each unsaturated fatty acid multiplied by the number of double bonds) and the ratio of fatty acids with 16 carbons and 18 carbons (C16:C18) were calculated. To test whether the fatty acid profiles were affected by the glyphosate concentration and temperature, each fatty acid profile was compared using a two-way ANOVA with the temperature and concentration as independent variables. The Shapiro–Wilk test was used to confirm the normal distribution of data, and the Bartlett test was used to check for homogeneity of variance. A post hoc test for multiple comparisons was conducted using Tukey’s test (*p* < 0.05). All the statistical data analyses were performed using SAS software (version 9.4; SAS Institute Inc., Cary, NC, USA). In addition, to visualize the differences in the fatty acid profiles across different glyphosate concentrations, principal component analysis (PCA) was conducted to evaluate the effects of glyphosate on the fatty acid composition. A permutational multivariate analysis (PERMANOVA) using the Euclidean distance as a similarity index was performed using the Adonis function in the Vegan package [40] of RStudio version 1.2.5001 [41].

## 3. Results

### 3.1. Toxicity Test

**Figure 1:**
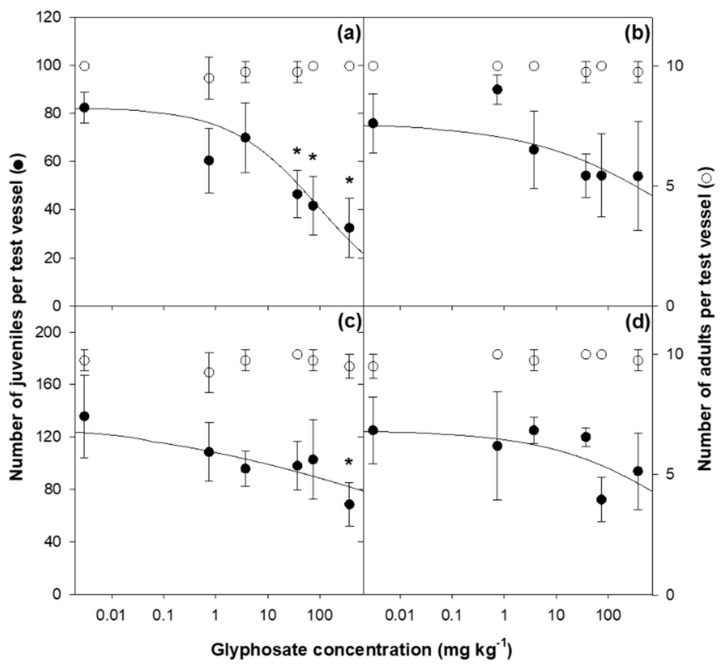
The number of surviving adults (○) and produced juveniles (●) of *Allonychiurus kimi* (Lee) after 28 d of exposure to glyphosate in OECD artificial soil at different temperatures (20 °C and 25 °C) and aging times (0 day and seven days); (**a**) 0 day of aging at 20 °C. (**b**) Seven days of aging at 20 °C. (**c**) 0 day of aging at 25 °C. (**d**) Seven days of aging at 25 °C. The data are presented as the mean ± standard deviation (per test vessel; *n* = 5). The solid lines are the number of juveniles estimated by fitting the data to the logistic model proposed by Haanstra et al. (1985). Asterisks indicate significant differences from each control (Dunnett’s post hoc test; *p* < 0.05).

The mean adult mortality rates in the controls (two for temperature and two for aging time) were all less than 10%, and no significant difference was detected between the controls (*p* > 0.05) (Figure 1). However, regardless of the aging time, the mean juvenile production per vessel in the control at 25 °C (135.8 ± 31.4 for 0 d and 125.0 ± 25.5 for seven days) was always significantly higher than that observed at 20 °C (82.5 ± 6.5 and 76.0 ± 12.3, respectively) (*p* < 0.05). The coefficient of variation calculated for the number of juveniles was less than 30% in all controls.

In all of the treatments, the adult survival within the entire test concentration range was not significantly different from that of the corresponding control, thereby indicating that glyphosate did not have a negative effect on adult survival (Figure 1). Thus, the median lethal concentration (LC_50_) for adults could not be determined because all of the mortality values were below 50%. Meanwhile, juvenile production in all of the treatments was reduced in a concentration-dependent manner, but a significant difference in juvenile production from that of the control was observed only at 0 d of aging. At 20 °C with 0 day of aging, the EC_50_ value was 93.5 mg kg^−1^ and the NOEC and LOEC values were 3.7 mg kg^−1^ and 37.1 mg kg^−1^, respectively (Figure 1 and Table 1). In the test at 25 °C with 0 day of aging, a significant difference from the control was observed only at the highest treatment concentration (370.5 mg kg^−1^); thus, the EC_50_ values could not be estimated. In the seven-day aging test at both temperatures, no significant difference from the control was observed even at the highest concentration, thereby indicating that the toxicity of glyphosate to *A. kimi* was reduced as the aging time and temperature increased.

### 3.2. Fatty Acid Composition

The TCLs in *A. kimi* adults were extracted 28 days after exposure to glyphosate at 20 °C and 25 °C. At both temperatures, the TCL concentration per individual was lower in the glyphosate treatments than in the control, but no significant difference was observed between the treatments (Table 2). The TCL concentration in the control was 6.90 ± 1.10 nmol ind−1 and 8.20 ± 4.40 nmol ind^−1^ at 20 °C and 25 °C, respectively, but that in the treatments ranged from 3.10 ± 0.60 nmol ind^−1^ to 3.80 ± 1.90 nmol ind^−1^.

Six fatty acids with a carbon chain length of 16 to 20 were identified as palmitoleic acid (16:1ω7), palmitic acid (16:0), linoleic acid (18:2ω6,9), oleic acid (18:1ω9), stearic acid (18:0), and arachidonic acid (20:4ω6,9,12,15). Each fatty acid composition is presented on a percentile scale in Table 2. Regardless of the temperature and glyphosate concentration, the most dominant fatty acids were oleic acid (39.65% to 44.63%) and palmitic acid (21.37% to 24.83%). The compositions of stearic acid and linoleic acid were very similar, ranging from 12.54% to 15.75% and 12.50% to 14.90%, respectively.

The two-way ANOVA results indicated that the proportions of palmitic acid, stearic acid, and arachidonic acid were significantly affected by the glyphosate concentration (*p* < 0.05), but no temperature effect on the six fatty acid profiles was observed (Table 3). The proportion of stearic acid, a long-chain saturated fatty acid, increased as the glyphosate concentration increased at 20 °C and 25 °C, but that of arachidonic acid, a polyunsaturated fatty acid (PUFA), decreased as the concentration increased and was not detected at the highest concentration at both temperatures. In addition, a significant interaction between the glyphosate concentration and temperature was detected in palmitoleic acid and oleic acid, but no significant differences in the proportion of each fatty acid were observed, except for palmitoleic acid, which was undetected at the highest concentration at 25 °C (Table 2).

**Figure 2:**
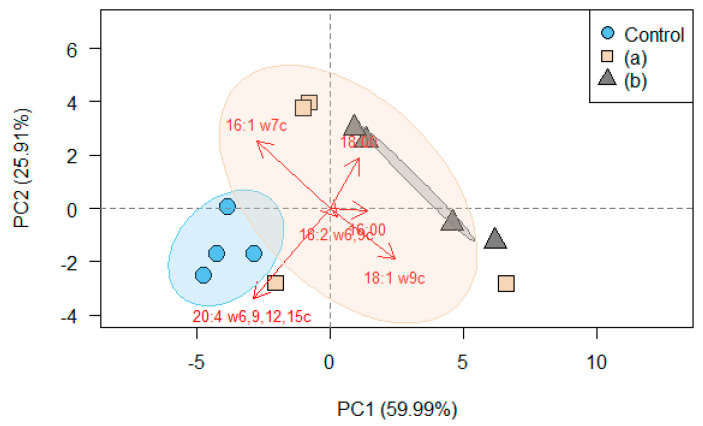
Biplot of principal component analysis (PCA) based on the fatty acid profiles (%) of *Allonychiurus kimi* (Lee) adults after 28 d of exposure to glyphosate at concentrations of 0.0 (control), 37.1 (a), and 370.5 (b) mg kg^−1^ with a 95% confidence ellipse. The arrows indicate the direction and magnitude at which each variable contributes to the variation between the treatments.

Owing to the increase in C18 and saturated fatty acids (stearic acid) and the decrease in C16 and unsaturated fatty acids (arachidonic acid and palmitoleic acid) due to glyphosate treatment, the UI and C16:C18 ratio at 25 °C decreased significantly as the glyphosate concentration increased. This was also evident from the biplot of the PCA, which clearly separated the fatty acid profiles exposed to different glyphosate concentrations according to the changes in the proportions of stearic acid and arachidonic acid (Figure 2). The PERMANOVA results also revealed significant influences of glyphosate application (*F* = 3.45; *p* = 0.02) on the fatty acid profiles.

### 3.3. Glyphosate Degradation

The residual glyphosate concentration in the soil decreased exponentially over time at both temperatures. The lag phase was not observed in the initial degradation state and after two days of incubation, and the residual glyphosate concentration at 20 °C (51.8 ± 9.0 mg kg^−1^) was significantly higher than that at 25 °C (27.5 ± 1.2 mg kg^−1^) (*p* < 0.05). At the end of the experiment, the residual glyphosate concentration at 20 °C was 2.2 ± 1.1 mg kg^−1^, but no glyphosate was detected at 25 °C, thereby indicating that glyphosate was rapidly degraded as the temperature increased.

Because the χ^2^ error values for the SFO model were smaller than the validity criteria (15%) and their visual fits and residual plots were acceptable [38], the SFO model was used to estimate the DT_50_ and DT_90_ values (Table 4). The DT_50_ and DT_90_ values at 20 °C (2.38 day and 7.91 day, respectively) were longer than those at 25 °C (1.69 day and 5.63 day, respectively).

## 4. Discussion

Glyphosate is known to be toxicologically and environmentally safe because of its unique mode of action and rapid degradation in the environment [1]. However, this study showed that glyphosate application can have a negative impact on the reproduction of *A. kimi,* and the impact decreased as the temperature and aging time increased. The fatty acid composition of *A. kimi* adults changed even at a lower concentration, at which reproduction was not impaired. Several studies have shown that changes in the response to toxic chemicals at the sub-individual level can lead to changes in the ecological fitness of individuals [25,26]. Thus, the significant changes in the fatty acid compositions suggest that glyphosate could have a chronic effect on *A. kimi* adults, and a long-term study is necessary to assess the ecotoxicological risk of glyphosate in the field. To the best of our knowledge, this is the first study to investigate the effects of temperature and aging time on the toxicity of glyphosate to soil organisms, including the biochemical effects at the sub-individual level.

The toxicity of pesticides may vary depending on the amount of pesticides which can change with the exposure temperature in the soil [19,42]. Wu and Nofziger (1999) [43] emphasized the importance of temperature for pesticide degradation in soil by reporting that temperature-dependent half-life models can predict atrazine residues in soil more accurately than constant half-life models. In this respect, the decrease in the toxicity of glyphosate to *A. kimi* as the temperature increased in this study (Figure 1) might have been attributed to the significant differences in the residual concentration. These results are comparable to previous findings that increased pesticide degradation with the increase in temperature can decrease the toxicity to various soil organisms such as mites and earthworms, including collembolan species [19,42]. Jegede et al. (2017) [19] reported that in a study on the toxicity of deltamethrin to *F. candida* in OECD artificial soil, as the temperature increased from 20 °C to 26 °C, the EC_50_ value for reproduction increased from 2.77 mg kg^−1^ to 12.85 mg kg^−1^. During pesticide degradation, metabolites are produced, which may be more reactive than the original chemical or may be inactive. If the metabolites of pesticides are more toxic than the parent compounds, the toxic effects could be increased with degradation [44]. In the case of glyphosate, the major metabolite in soil is aminomethylphosphonic acid, which has very low toxicity to various soil animals [14,45]. Thus, the glyphosate toxicity in soil would have been mainly determined by the remaining residual concentrations and not by the metabolites. In addition, the pesticide uptake rate in relation to temperature may also lead to changes in pesticide toxicity [19,42]. Because glyphosate has strong hydrophilicity and rapid degradation in soil [5,46], it is believed that the increase in uptake with temperature might not have led to increased bioaccumulation in this study.

The aging process alters the fate of chemical contaminants, thereby resulting in changes in their toxicity [47,48]. In general, studies on the pesticide aging process in soil have focused on the decrease in the bioavailability of recalcitrant substances by sequestration into soil nanopores and organic matter matrixes [48,49]. For example, Robertson and Alexander (1998) [48] reported that the toxicity of dieldrin, a persistent insecticide, to fruit flies (*Drosophila melanogaster*) and cockroaches (*Blattella germanica*) was decreased by sequestration into the soil as aging increased, although 92.1% of the initial concentration of dieldrin remained in the soil after 270 day of aging. However, in our study, the residual glyphosate concentration decreased as the aging period increased regardless of the temperature, and the glyphosate toxicity was only significant at 0 day of aging at both 20 °C and 25 °C (Figure 1), thereby indicating that the decreased toxicity as the aging period increased was closely related to the remaining glyphosate concentration. Seven days after the glyphosate treatment (7 days of aging), nearly 90% of the initial glyphosate concentration was degraded (DT_90_ = 7.91 day at 20 °C and 5.63 day at 25 °C; Table 4) and only 10% of the initial concentration was exposed. The concentration was further degraded during the 28-day exposure period. These results suggest that the aging effect of glyphosate, which breaks down quickly and easily in the soil, can be mainly determined by the residual amount remaining in the soil after aging.

Our results showed that glyphosate toxicity in soil is mainly determined by temperature-dependent degradation. Therefore, to assess the ecologically relevant risk of glyphosate in fields in which the temperature fluctuates, it is essential to understand the temperature-dependent half-life of glyphosate, which was ignored in the standard toxicity test conducted under constant environmental conditions (i.e., 20 ± 2 °C) [43]. The temperature might have been crucial in determining the toxicity in this study because glyphosate is known to be degraded primarily by microorganisms in the soil [1,3], and the microbial activity is temperature-dependent [50]. Bento et al. (2016) [51] compared the degradation of glyphosate in soils with and without microorganisms. The difference in the degradation rate of glyphosate was due to the temperature-dependent microbial activity. Hofman et al. (2014) [52] reported that the degradation rate of biodegradable phenanthrene in artificial soil can vary depending on the amount of indigenous microorganisms through a study using 10 OECD artificial soils prepared in different laboratories. Therefore, further studies on the effects of temperature on the microbial activity in soil, as well as other environmental factors such as soil humidity, soil texture, and organic matter content, which may affect the soil microbial activity and microbial composition, would provide a more realistic and accurate estimate of glyphosate toxicity [53,54].

In evaluating the risks of pesticides, identifying the effects of pesticides on organisms at the sub-individual level is important because pesticides may not only affect the survival or reproduction of organisms, but also impair the ecological fitness of individuals through sublethal effects [25,26,28]. Our study using fatty acids as a biomarker confirmed that *A. kimi* can be biochemically affected by glyphosate treatment even at the concentrations at which no glyphosate-induced adult mortality and juvenile reproduction were observed (Figure 2 and Table 2). The decrease in the proportion of arachidonic acid (20:4ω6,9,12,15) by the application of glyphosate might have been attributed to the loss of the ability to synthesize C20 PUFAs because C20 PUFAs in collembolan species are usually obtained by synthesizing precursors [55]. Given the function of arachidonic acid, which is the main precursor of eicosanoids and mediates physiological processes such as reproduction, immunity, and metabolism in mammals and insects [56,57], the decrease in the ability to synthesize C20 PUFAs can cause harmful effects at the individual level. In contrast to arachidonic acid, the proportion of stearic acid (18:0) increased as the glyphosate concentration increased (Table 2). Similar to our findings, increases in stearic acid in organisms have been reported in response to stressful conditions, such as exposure to toxic substances or high salinity [58,59]. Although it is still unknown how the increase in stearic acid is induced, considering the role of stearic acid, which is also a precursor of eicosanoids [60], it might have increased in response to the decrease in arachidonic acid for eicosanoid synthesis. Further studies on the complementary roles of arachidonic acid and stearic acid in the synthesis of eicosanoids may provide a better understanding of changes in the fatty acid composition of organisms in stressful situations. Although the fatty acid composition of *A. kimi* exposed to glyphosate was investigated only for the temperature effects in this study, these changes related to the physiological processes of an organism can have harmful effects at the individual level or higher in the long-term and continue through generations [27]. The observed changes in the fatty acid composition of *A. kimi* indicated that fatty acid composition is a useful tool for detecting the deleterious effects of glyphosate at the cellular level. Furthermore, given the essential role of fatty acids in organisms [30], there may be potential negative effects of glyphosate application that have not yet been revealed.

Globally, more than 8.6 billion kg of glyphosate has been applied and accumulated in the soil since 1974 [4]. The maximum predicted environmental concentration (PEC) of glyphosate in soil accumulated for 10 y was estimated to be 6.62 mg kg^−1^, assuming that glyphosate was applied at the maximum application rate (4.32 kg glyphosate ha^−1^) once a year [61]. However, considering the increase in the frequency and amount of glyphosate applications [5], the actual glyphosate concentrations in soil could be much higher than those previously estimated. Recently, Karasali et al. (2019) [62] reported that the glyphosate residues in Greek soils was mostly observed lower than the PEC, but much higher residual concentration than the PEC, ranged from 7.2 – 40.6 mg kg^−1^, was detected in urban area and olive groves. In the present study, the effects of glyphosate exposure to *A. kimi* near the PEC level was not evaluated; however, the changes in the fatty acid composition of *A. kimi* at the glyphosate concentrations that can be frequently observed in the environment indicate that glyphosate can have a potential negative impact on the soil ecosystem. Thus, to better understand the effect of glyphosate in the soil on the soil ecosystem, additional studies are needed on the changes in glyphosate toxicity with environmental factors such as humidity and soil texture over long-term time scales.

## 5. Conclusions

Glyphosate have been known to be toxicologically and environmentally benign, but our study showed that glyphosate can have negative effects on the juvenile production of *A. kimi* depending on the temperature and aging time. Glyphosate toxicity is largely determined by the amount of residue in the soil, which is dependent on the soil temperature. Thus, an understanding of the temperature-dependent half-life of glyphosate in various field soils in which the temperature fluctuates considerably is important to assess the ecologically relevant risk under field conditions. No glyphosate-induced adult mortality was observed in any of the treatment conditions, but significant changes in the fatty acid composition of *A. kimi* adults, which play a critical role in energy storage, cell structure, and regulatory physiology, were observed, thereby suggesting that glyphosate could have adverse effects on *A. kimi* at the sub-individual level. Considering the increasing application rate and repeated application of glyphosate in fields [5], glyphosate concentrations higher than the PEC could be present in the soil. If *A. kimi* is exposed to glyphosate for a long time above the PEC, then the ecological fitness of an individual may also be affected. Therefore, it is necessary to investigate the long-term impacts of glyphosate by conducting higher-tier tests, such as multigenerational tests.

## Figures and Tables

**Table 1:** Median effective concentration (EC50) with corresponding 95% confidence intervals, no observed effect concentration (NOEC), and lowest observed effect concentration (LOEC) of glyphosate on the reproduction of *Allonychiurus kimi* (Lee) after 28 days of exposure to glyphosate in artificial soil at two temperatures (20 °C and 25 °C) and aging times (0 day and seven days) estimated using the logistic model presented by Haanstra et al. (1985).

Temperature (°C)	Aging Time (d)	EC_50_	NOEC ^b^	LOEC ^b^
20	0	93.5 (25.9–161.2)	3.7	37.1
	7	- ^a^	-	-
25	0	-	74.1	370.5
	7	-	-	-

^a^ The EC_50_ could not be determined because the observed reproduction values were over 50%, even when exposed to the highest concentration tested (370.5 mg kg^−1^). ^b^ The NOEC and LOEC were determined using Dunnett’s post hoc test (*p* < 0.05).

**Table 2:** Proportions of fatty acids (mean ± standard deviation) in *Allonychiurus kimi* (Lee) adults after 28 day of exposure to glyphosate (0.0, 37.1, and 370.5 mg kg^−1^) at 20 °C and 25 °C.

		20 °C			25 °C		
		Glyphosate Concentration (mg kg^−1^)			Glyphosate Concentration (mg kg^−1^)		
Fatty Acids		0.0	37.1	370.5	0.0	37.1	370.5
Palmitoleic acid	16:1ω7	5.77 ± 1.06	5.60 ± 3.68	5.48 ± 0.17	5.16 ± 0.18	6.72 ± 1.34	**0** **.00 ± 0.00**
Palmitic acid	16:0	21.37 ± 1.15	24.31 ± 0.43	23.79 ± 0.26	22.87 ± 0.71	22.84 ± 0.11	24.83 ± 1.30
Linoleic acid	18:2ω6,9	14.90 ± 0.95	13.41 ± 1.98	13.60 ± 0.93	12.50 ± 1.07	13.03 ± 0.81	13.70 ± 0.57
Oleic acid	18:1ω9	39.65 ± 0.07	44.63 ± 2.96	41.40 ± 1.11	40.18 ± 0.86	39.54 ± 1.68	43.46 ± 1.40
Stearic acid	18:0	12.84 ± 0.39	12.54 ± 1.87	**15.75 ± 0.11**	13.38 ± 0.17	**14.99 ± 0.** **37**	**15.49 ± 0.** **30**
Arachidonic acid	20:4ω6,9,12,15	5.49 ± 1.58	2.51 ± 3.55	0.00 ± 0.00	5.93 ± 0.47	**0** **.00 ± 0.00**	**0** **.00 ± 0.00**
Total cellular lipids (nmol·ind^−1^)		6.90 ± 1.10	3.80 ± 1.90	3.70 ± 1.20	8.20 ± 4.40	3.70 ± 0.20	3.10 ± 0.60
C16:C18 ^a^		0.40 ± 0.04	0.39 ± 0.08	0.41 ± 0.00	0.42 ± 0.01	0.44 ± 0.00	**0.34 ± 0.** **01**
Unsaturation index ^b^		0.97 ± 0.07	0.84 ± 0.11	0.74 ± 0.01	0.94 ± 0.03	**0.72 ± 0.** **05**	**0.71 ± 0.** **03**

The bold numbers indicate a significant difference from the control within the same temperature treatment (Dunnett’s post hoc test; *p* < 0.05). ^a^ The ratio of fatty acids with 16 carbons and 18 carbons. ^b^ The unsaturation index was calculated as described by Haubert et al. (2004).

**Table 3:** Two-way analysis of variance results on the effects of glyphosate and temperature on the individual fatty acid contents in *Allonychiurus kimi* (Lee) adults after 28 day of exposure to glyphosate.

Fatty Acids		Glyphosate (G)		Temperature (T)		G × T	
		*F*	*p*	*F*	*p*	*F*	*p*
Palmitoleic acid	16:1ω7	3.02	0.12	0.76	0.42	8.00	**0.02**
Palmitic acid	16:0	7.91	**0.02**	0.64	0.45	4.05	0.08
Linoleic acid	18:2ω6,9	0.21	0.82	1.78	0.23	1.35	0.33
Oleic acid	18:1ω9c	2.90	0.13	0.81	0.40	5.53	**0.04**
Stearic acid	18:0	9.41	**0.** **01**	3.72	0.10	2.82	0.14
Arachidonic acid	20:4ω6,9,12,15	14.14	**0.01**	0.78	0.41	1.05	0.41
C16:C18 ^a^		1.15	0.38	0.00	0.98	3.03	0.12
Unsaturation index		11.74	**0.01**	2.28	0.18	0.29	0.76

The bold numbers indicate a significant effect of the factor on individual fatty acids (*p* < 0.05). ^a^ The ratio of fatty acids with 16 carbons and 18 carbons.

**Table 4:** Degradation kinetics parameter (*k*), DT*_x_* values, and χ^2^ error for glyphosate fitted with the single first-order model in soil at 20 °C and 25 °C.

Temperature (°C)	*k*	DT_50_ (d)	DT_90_ (d)	χ^2^ Error (%)
20	0.291	2.38 (1.74–3.02)	7.91 (5.79–10.05)	9.55
25	0.409	1.69 (1.27–2.11)	5.63 (4.23–7.01)	14.02

DT*_x_* values indicate the time required for the initial concentration to decrease *x*%. The degradation kinetics of glyphosate were investigated in soil contaminated with 370.5 mg kg^−1^ of glyphosate at both temperatures.

## Data Availability

Data is contained within the article.
